# Retraction: Pressure regulated basis for gene transcription by delta-cell micro-compliance modeled in silico: Biphenyl, bisphenol and small molecule ligand models of cell contraction-expansion

**DOI:** 10.1371/journal.pone.0249385

**Published:** 2021-03-25

**Authors:** The PLOS ONE Editors

After this article [1] was published, the corresponding author contacted *PLOS ONE* to request corrections. The author advised that he did not intend for [1] to be published in its current online form, and that the article should be retracted in the event that the requested revisions could not be incorporated into the published version of the article.

The requested changes involve substantial revisions to the rationale, methodology, results and conclusions reported in the article text, as well as changes to Tables 3, 6, 7, 8, 10, 11, 12, and Figs 2 and 3. The *PLOS ONE* Editors determined that the requested revisions go beyond what is suitable for a Correction per the journal’s editorial standards and would instead necessitate a full re-review of an updated manuscript.

In reviewing the correction request, the *PLOS ONE* Editors identified additional concerns with the article, including:

- A specific scientific rationale for the selection of representative small molecules and genes was not provided, raising questions about whether the results presented are applicable beyond the compounds studied.
- The methods were not described in sufficient detail to clearly relay the study design and enable other researchers to interpret the results or reproduce the analyses.
- The article did not report adequate validation of the *in silico* model to support the reported claims about gene transcription regulation.

In light of these additional issues, the *PLOS ONE* Editors determined that the published article does not meet the journal’s publication criteria and there are concerns about the validity of the reported results and conclusions.

Based on the outcome of the editorial assessment and in line with the author’s request, the *PLOS ONE* Editors retract this article. We regret that the concerns about this work were not addressed prior to the article’s publication.

In response to the retraction decision, HS expressed an intention to address the above issues in a future submission of this work.
